# Impact of Cyberbullying on Adolescent Mental Health in the midst of pandemic – Hidden Crisis

**DOI:** 10.1192/j.eurpsy.2022.1096

**Published:** 2022-09-01

**Authors:** Y. Deol, M. Lashai

**Affiliations:** Icahn School of Medicine at Mount Sinai, Psychiatry, Elmhurst, United States of America

**Keywords:** social media, cyberbullying, adolescent mental health

## Abstract

**Introduction:**

Cyberbullying has become more prevalent with the increased use of social media among younger population. It is more harmful than traditional bullying as it can happen at any time, has a much wider audience, and can invade personal space. YouTube, Instagram and Snapchat are the most popular online platforms among teens. The victims of cyberbullying can present with social anxiety (41%), depression (37%), suicidal thoughts (26%) among many others (self-harm, substance use, etc). In the past year, these numbers have significantly risen due to switch to virtual learning due to the pandemic, hence the risk of exposure to cyberbullying has risen.

**Objectives:**

To study the impact of cyberbullying on Adolescent Mental health

**Methods:**

A review of articles (2016-2021), was done using PubMed and Google scholar focusing on impact of cyberbullying in children and young adults.

**Results:**

John et al group showed that both victims [OR- 2.10 (95% CI 1.73-2.55)] and perpetrators [ OR 1.21 (95% CI 1.02-1.44)] have increased risk of exhibiting suicidal behaviors. Kwan I et al group showed a negative association between cyberbullying and mental health. One study suggested that during the pandemic there has been increased online perpetrators due to increased amount of fear and anger which has projected in the form online aggression.

**Conclusions:**

There is an increase in prevalence of cyberbullying with young population spending more time on internet and social media. Psychoeducation of parents and mental health experts is needed to recognize early warning signs in order to take steps for early intervention.

**Disclosure:**

No significant relationships.

